# Running into the Lyme-light: a retrospective cross-sectional study of tick bites and Lyme disease prevalence, incidence, and prevention in hill runners, Scotland, UK.

**DOI:** 10.1186/s12889-025-26181-8

**Published:** 2026-01-13

**Authors:** Mabel Balfour, Rita Ribeiro, Harriet Auty, Alicia Heath

**Affiliations:** 1 School of Public Health, Imperial College London, London, UK; 2 School of Biodiversity, One Health and Veterinary Medicine, College of Medical, Veterinary and Life Sciences, University of Glasgow, Glasgow, UK

**Keywords:** *Borrelia burgdorferi*, Hill running, *Ixodes ricinus*, Prevention, Tick-borne infection, Zoonoses

## Abstract

**Background:**

Lyme disease (LD) incidence is increasing globally, driven by changes to habitat and human-vector interactions. Effective prevention relies on local and individual-level understanding of risk and ecosystem interactions. Despite frequent exposure to tick habitats, little is known about tick bites and LD risk among hill, trail, and mountain runners. This study aimed to evaluate the period prevalence of tick bites and incidence of LD in hill runners in Scotland, UK and examine preventive behaviours.

**Methods:**

A cross-sectional retrospective survey of Scottish hill runners was conducted in June-July 2024. Hill runners aged 18 + resident in Scotland for the 12 months preceding the survey were eligible. Period prevalence was calculated as the proportion of hill runners with at least one self-reported tick bite in the 12 months preceding the survey. Treated suspected LD incidence was calculated for all study participants and those registered in competitive hill races. Multiple logistic regression was used to estimate odds ratios (ORs) and 95% confidence intervals (CIs) of having a tick bite, for variables including age, sex, running hours per week, proportion of running that is hill running, other outdoor activity hours per week, frequency of insect repellent use, full leg and arm cover use, and full body tick checks.

**Results:**

212 hill runners (56.1% male) completed the survey. Period prevalence of at least one tick bite was 82.5% (95%CI 76.76–87.40). Incidence of treated suspected LD for the 212 study participants was 4245.3 per 100,000 (95%CI 1941.21-8058.87), and the minimum estimate in 4668 registered competitive hill racers was 150.0 per 100,000 per year (95%CI 60.29-309.97). Most hill runners reported never/infrequently wearing full leg cover (67.9%), arm cover (58.0%), or using insect repellent (74.5%). Regularly/almost always conducting a tick check was associated with higher odds of at least one tick bite (OR 8.52, 95%CI 3.29–22.90, *p* < 0.001), whereas regularly/almost always wearing full leg cover was associated with lower odds (OR 0.35, 95%CI 0.13–0.89, *p* = 0.029), versus never/infrequently.

**Conclusions:**

A high reported period prevalence of tick bites and incidence of treated suspected LD among surveyed hill runners in Scotland aligned with the low adherence to tick bite prevention behaviours in this population. Improving adherence with leg cover recommendations may lower the risk of tick bites. Raising awareness of tick bite risk and prevention may benefit hill runners internationally as the sport grows.

**Supplementary Information:**

The online version contains supplementary material available at 10.1186/s12889-025-26181-8.

## Background

The incidence of Lyme disease (LD), caused by the bacteria *Borrelia burgdorferi*, is rising across Europe [1], including within the United Kingdom (UK) [2, 3]. This tick-borne infection can cause significant health issues if left untreated, including arthritis, carditis and neurological symptoms [4]. Diagnosis can be made clinically via a localised erythema migrans rash specific to the infection or through serological testing [4–6]. A myriad of symptoms, often non-specific, makes LD difficult to identify [4, 7], and the occurrence of persisting symptoms even after antibiotic therapy [8] means prevention is key to public health management. Effective targeted mitigation relies on accurate surveillance and a thorough understanding of transmission routes and risk factors [9–11].

Multiple indicators from national surveillance [2] and primary care research [6] suggest that Scotland has the highest incidence of LD in humans in the UK. Differences in estimates across the UK and internationally are likely to be multifactorial [12]. In part, differences in LD incidence estimates are related to variations in case definitions, diagnostic practices and data collection [12]. Surveillance worldwide is limited by the challenges of accurate diagnosis, as well as a lack of routine serological testing in clinical practice [5, 6, 12, 13], which in the UK is consistent with national guidelines stating that patients with erythema migrans should be empirically treated [4]. Reported incidence rates in the UK are based on laboratory-diagnosed cases only and are likely underestimates given the lack of mandatory reporting of clinical cases [2, 14, 15]. Furthermore, access to accurate clinical diagnosis data is limited even within healthcare datasets due to the inconsistency of clinical case and antimicrobial recording [6, 15]. Variation in environmental tick abundance, driven by differences in climate, habitat suitability, and host distribution [10, 11], also contributes to the likelihood of vector interactions. Human exposure to tick habitats [6, 16], including long grasses, forests, moorlands, and even urban parklands and gardens [5, 17–19], also differs. Changing vector distribution and non-native species introduction [20], and the spread of further pathogens, such as tick-borne encephalitis virus (TBEV) in native UK tick populations [21, 22], add to the complexity and importance of understanding the risk of tick-borne disease. Improving surveillance can help untangle reasons for differences in incidence across the UK and abroad [10, 11, 13]. Enhancing epidemiological evidence of local high-risk populations and risk factors for tick bites will enable more appropriate targeted preventive strategies for reducing LD burden [10, 11]. However, a granular understanding of differences in regional burden [1] and high-quality evidence for effective prevention strategies [23] are currently lacking.

Previously identified high-risk groups for tick-borne diseases include people in forestry and farming occupations, as well as those participating in recreational outdoor activities [21, 24, 25]. Several online surveys have assessed tick bite frequency and found a strong association between the frequency of running and walking in forested areas in Scandinavia [26] and on marked trails in the USA, and acquiring tick bites [27]. In the latter study, running and walking had a higher risk for tick bites compared to orienteering and other outdoor activities [27]. However, these studies are limited by a reliance on self-reporting, and the combination of activities assessed makes it difficult to pinpoint the risk associated with any specific activity. Moreover, variability in the duration and frequency of individual activities were not taken into account. Nevertheless, these previous studies demonstrate how citizen science can lead to informative datasets containing key information, such as the spatial distribution of tick species and bite acquisition in populations previously poorly studied, and have helped to identify potential groups and activities with increased risk of tick bites [20].

Several international studies have demonstrated the high risk of tick bites in orienteering activities, despite full body coverage being required during competition due to these risks [17, 28–30], aligning with the behavioural interventions recommended by the National Health Service (NHS) [31]. Hill (or fell) running in the UK is similar to international trail and mountain running, but like orienteering, it is a sport that often requires off-path running in tick habitat [17, 30, 32, 33], including in areas considered high risk for LD, such as the Scottish Highlands [4]. Only one previous survey has examined tick bites in hill runners, focusing on participants of a two-day event in Scotland in 2014, with 8.5–13.8% of runners reporting tick bites acquired during the event [34]. Variability in clothing was observed, with many wearing shorts and short-sleeved tops [34]. As no attire requirements are placed on hill runners, it is hypothesised that there may be differences in body cover and thus differences in tick bite and LD risk, compared to orienteers. These previous studies demonstrate that tick bite reports at mass participation sporting events can be useful indicators of tick activity in the environment [17, 34]. However, there is a lack of data on the prevalence of tick bites and the impact of tick-borne disease outside of specific events among individuals exercising in high-risk terrain.

This study aimed to evaluate: (1) the period prevalence of tick bites, and (2) the period prevalence and incidence of reported treated suspected LD diagnoses in hill runners in Scotland, UK; (3) examine whether tick bite prevention behaviours are practised by this group; and (4) evaluate whether these behaviours are associated with tick bites. Gaining a better understanding of the period prevalence of tick bites and LD incidence, and the uptake and effectiveness of prevention behaviours in the hill running population in Scotland, will enable appropriate targeted preventive action. As hill, trail and mountain running become increasingly popular internationally, there is scope for research in this field to have wider impacts for these communities as the sphere of regulation and interest expands.

## Methods

### Design

A retrospective, cross-sectional digital survey of hill runners was conducted in Scotland, UK. A 32-question questionnaire was developed for this study (Additional file 1), which captured hill running activity, including competitive racing and metrics of time spent running, with divisions by time spent over forestry, long grass and moorland (‘hill terrain’). Other questions included participant demographics, tick bite and LD history and related experiences, including antibiotic use. Specific questions explored tick bite prevention behaviours recommended by NHS Scotland [31].

### Recruitment

The number of persons taking part in hill running and identifying as a hill runner in Scotland is unknown; however, 4668 individual entrants (recorded by the Scottish Hill Runners (SHR)) took part in SHR-affiliated races in the 12 months preceding the survey. Potential participants were identified and recruited via email invitations and social media recruitment campaigns administered by SHR to their members across Scotland (an estimated 550 in 2023). A recruitment poster and advertisement were also placed on Hill Runner social media pages (Facebook and Instagram). The survey was distributed digitally via Qualtrics survey software (Provo, UT). Volunteers self-selected to participate after reading the participant information sheets and signed a consent form at the start of the digital survey. Recruitment and survey completion took place from June to July 2024.

### Inclusion and exclusion criteria

Only persons residing in Scotland for the 12 months preceding recruitment were eligible for inclusion to align with the period under investigation and minimise the confounding effects of geographic region, where recommendations and risk may differ. Participants were asked to confirm this, as well as specifying their county of residence. This ensured that findings could be attributed to running primarily in Scotland. A question regarding tick bites acquired outside of Scotland enabled investigation of risk attributable elsewhere.

Participants self-identified as hill runners based on the SHR definition [35], assuming they could accurately identify the terrain they typically ran on during the study period. Hill running was defined as running over hills, moors and mountains. Additional questions enabled further clarity on the amount of training, for more accurate estimation of the prevalence of tick bites and LD stratified by time spent running over higher risk terrains, as well as competitive hill running to estimate the impact of competition on behaviours that may modify risk.

Persons under the age of 18 years were excluded to align with restricted age groups in hill racing [35]. Participants who were ineligible based on inclusion and exclusion criteria were directed out of the survey and thanked for their interest in participating.

### Data processing

Participants were grouped based on their competitive hill racing history over the past 12 months, to identify those who had taken part in SHR-affiliated races or other events they defined as a hill race. This information was included to explore potential differences in adherence to prevention behaviours among competitive hill runners compared with non-competitive hill runners. Preventive behaviours included the NHS recommendations of wearing full arm cover, full leg cover, applying insect repellent, and conducting full body tick checks, particularly between March to October [31]. The NHS recommendations generally align with increased tick activity and abundance in the environment, although it is acknowledged that seasonal tick activity may be changing [36]. The proportion of time complying with these behaviours between March and October, when on hill terrain (defined as heather, bracken, long grass and/or forestry), was divided into never/ infrequently, regularly, or almost always, as was the proportion of time running on hill terrain.

Respondents reported the number of tick bites they had incurred in the 12 months preceding the survey (none, 1–5, 6–10, 11–15, 16–20, ≥ 21). For analyses, the main outcome was defined as having at least one tick bite in the 12 months preceding the survey (no/yes). A secondary outcome was treated suspected LD diagnosis in the preceding 12 months, derived from reported diagnosis of LD by a healthcare professional (no/yes) or reported erythema migrans (no/yes), and treated with a course of antibiotics (no/yes).

### Statistical analysis

Differences between categorical variables under investigation were tested with a Fisher exact test and Pearson Chi-square test, and continuous variables with a Wilcoxon rank sum test (ɑ=0.05). Period prevalence of tick bites was calculated as the proportion of survey participants reporting at least one tick bite in the 12 months preceding the survey, and separately, in their lifetime prior to the 12-month survey period. Period prevalence and incidence of treated suspected LD was calculated using the number of participants as the denominator. Incidence of treated suspected LD was also calculated for geographical areas where participants reported a diagnosis. Acknowledging that survey participation may be biased towards individuals who have previously been diagnosed with LD, an estimated minimum incidence of treated suspected LD in SHR registered competitive hill runners was calculated based on the subset of participants who reported taking part in SHR-affiliated races, using the reported total denominator of 4668 racers provided by SHR.

Multiple logistic regression modelling was conducted to assess the associations between all variables under investigation and the odds of having at least one tick bite in the 12 months preceding the survey. Variables evaluated included age (continuous), sex [37], hours per week of running (continuous) [17], the proportion of running that was hill running (continuous), hours per week of other outdoor activities, including occupational activities (continuous), frequency of using insect repellent [23, 38], and of wearing full arm cover, full leg cover and conducting full body tick checks (grouped as never/infrequently and regularly/almost always) [31]. Participants who gave implausible answers for average hours/week running (> 50 h/week) or taking part in other outdoor activity (> 100 h/week) were excluded from the regression analyses (*n* = 6). All variables included in the model were tested for multicollinearity using a variance inflation factor score, which was low (< 1.5) [39]. A McFadden’s pseudo-R-squared score was calculated to assess the proportion of variance in the outcome (having at least one tick bite) explained by the included variables. Dominance analysis for logistic regression [40] was conducted to analyse the relative importance of each variable. This was determined by comparing pairs of variables and assessing the additional contribution of each variable to the model.

All statistical analyses were conducted in R version 4.1.0 [41].

### Ethical considerations

Ethical approval was obtained from Imperial College London’s Research Governance and Integrity Team (Imperial College Research Ethics Committee reference 7030885). The study was conducted following the recommendations for physicians involved in research on human subjects adopted by the 18th World Medical Assembly, Helsinki 1964 and later revisions. All participants gave informed consent in the digital survey form and confirmed they were aged 18 years or over.

## Results

A total of 224 individuals responded to the survey. After exclusions due to residency outside Scotland (*n* = 9) and non-participation in hill running in the preceding 12 months (*n* = 3), 212 hill runners completed the survey, of which 56.1% (*n* = 119) were male and 43.9% (*n* = 93) female (Table 1). Individuals reporting participation in a SHR-affiliated race (*n* = 144) represented 67.9% of respondents, and 3.1% of the total number of 4668 racers registered in SHR-affiliated races in the 12 months preceding the survey.

**Table 1:** Summary of survey responses from 212 hill runners in Scotland

Variable	Overall, *N* = 212^*c*^	Tick bite in the 12 months preceding survey^*a*^			Treated suspected Lyme disease in the 12 months preceding survey^*b*^		
		No, *N* = 37^*c*^	Yes, *N* = 175^*c*^	*p*-value^*d*^	No, *N* = 197^*c*^	Yes, *N* = 9^*c*^	*p*-value^*e*^
Reported number of tick bites in preceding 12 months				**< 0.001**			0.610
None	37 (17.5%)	37 (100.0%)	0 (0.0%)		35 (17.8%)	0 (0.0%)	
1–5	59 (27.8%)	0 (0.0%)	59 (33.7%)		54 (27.4%)	3 (33.3%)	
6–10	31 (14.6%)	0 (0.0%)	31 (17.7%)		29 (14.7%)	1 (11.1%)	
11–15	30 (14.2%)	0 (0.0%)	30 (17.1%)		29 (14.7%)	1 (11.1%)	
16–20	12 (5.7%)	0 (0.0%)	12 (6.9%)		11 (5.6%)	1 (11.1%)	
≥ 21	43 (20.3%)	0 (0.0%)	43 (24.6%)		39 (19.8%)	3 (33.3%)	
Reported number of tick bites in lifetime prior to 12 month survey period				**< 0.001**			0.120
None	18 (8.5%)	18 (48.6%)	0 (0.0%)		17 (8.6%)	0 (0.0%)	
1–5	24 (11.3%)	12 (32.4%)	12 (6.9%)		24 (12.2%)	0 (0.0%)	
6–10	21 (9.9%)	3 (8.1%)	18 (10.3%)		20 (10.2%)	0 (0.0%)	
11–15	9 (4.2%)	1 (2.7%)	8 (4.6%)		6 (3.0%)	2 (22.2%)	
16–20	16 (7.5%)	0 (0.0%)	16 (9.1%)		16 (8.1%)	0 (0.0%)	
≥ 21	124 (58.5%)	3 (8.1%)	121 (69.1%)		114 (57.9%)	7 (77.8%)	
Sought medical attention for a tick bite in preceding 12 months				**0.048**			**< 0.001**
No	194 (91.5%)	37 (100.0%)	157 (89.7%)		188 (95.4%)	0 (0.0%)	
Yes	18 (8.5%)	0 (0.0%)	18 (10.3%)		9 (4.6%)	9 (100.0%)	
Antibiotics to treat tick bite in preceding 12 months				**0.033**			**< 0.001**
No	193 (91.0%)	35 (94.6%)	158 (90.3%)		187 (94.9%)	0 (0.0%)	
Yes	15 (7.1%)	0 (0.0%)	15 (8.6%)		6 (3.0%)	9 (100.0%)	
Not sure	4 (1.9%)	2 (5.4%)	2 (1.1%)		4 (2.0%)	0 (0.0%)	
Age (years)	40 (31, 49)	43 (31, 50)	40 (31, 48)	0.552	41 (31, 49)	34 (32, 37)	0.579
Sex				0.654			1.000
Female	93 (43.9%)	15 (40.5%)	78 (44.6%)		86 (43.7%)	4 (44.4%)	
Male	119 (56.1%)	22 (59.5%)	97 (55.4%)		111 (56.3%)	5 (55.6%)	
Competitive hill racing in the preceding 12 months				0.907			0.697
No	50 (23.6%)	9 (24.3%)	41 (23.4%)		45 (22.8%)	1 (11.1%)	
Yes	162 (76.4%)	28 (75.7%)	134 (76.6%)		152 (77.2%)	8 (88.9%)	
Hours per week of running	6.0 (4.2, 8.0)	6.0 (4.5, 8.0)	6.0 (4.5, 8.0)	0.877	6.0 (4.0, 8.0)	8.0 (7.0, 10.0)	0.054
Proportion of running that is hill running				0.764			0.669
Never or infrequently	32 (15.1%)	7 (18.9%)	25 (14.3%)		29 (14.7%)	2 (22.2%)	
Regularly	142 (67.0%)	24 (64.9%)	118 (67.4%)		133 (67.5%)	5 (55.6%)	
Almost always	38 (17.9%)	6 (16.2%)	32 (18.3%)		35 (17.8%)	2 (22.2%)	
Hours per week of other outdoor activity	5.0 (3.0, 8.0)	5.0 (3.0, 6.5)	5.0 (3.0, 10.0)	0.465	5.0 (3.0, 8.0)	6.0 (4.0, 15.0)	0.128
Insect repellent use				0.946			0.431
Never or infrequently	158 (74.5%)	27 (73.0%)	131 (74.9%)		149 (75.6%)	6 (66.7%)	
Regularly	41 (19.3%)	8 (21.6%)	33 (18.9%)		37 (18.8%)	2 (22.2%)	
Almost always	13 (6.1%)	2 (5.4%)	11 (6.3%)		11 (5.6%)	1 (11.1%)	
Full leg cover				0.135			1.000
Never or infrequently	144 (67.9%)	21 (56.8%)	123 (70.3%)		133 (67.5%)	7 (77.8%)	
Regularly	53 (25.0%)	11 (29.7%)	42 (24.0%)		50 (25.4%)	2 (22.2%)	
Almost always	15 (7.1%)	5 (13.5%)	10 (5.7%)		14 (7.1%)	0 (0.0%)	
Full arm cover				1.000			0.644
Never or infrequently	123 (58.0%)	22 (59.5%)	101 (57.7%)		115 (58.4%)	4 (44.4%)	
Regularly	81 (38.2%)	14 (37.8%)	67 (38.3%)		74 (37.6%)	5 (55.6%)	
Almost always	8 (3.8%)	1 (2.7%)	7 (4.0%)		8 (4.1%)	0 (0.0%)	
Full body tick check				**< 0.001**			0.645
Never or infrequently	29 (13.7%)	14 (37.8%)	15 (8.6%)		27 (13.7%)	2 (22.2%)	
Regularly	69 (32.5%)	12 (32.4%)	57 (32.6%)		66 (33.5%)	2 (22.2%)	
Almost always	114 (53.8%)	11 (29.7%)	103 (58.9%)		104 (52.8%)	5 (55.6%)	

^*a*^ Reported tick bite (at least one) in the 12 months preceding the survey

^*b*^ Reported treated suspected Lyme disease (either reported clinical diagnosis by healthcare professional or erythema migrans, treated with antibiotics) in the 12 months preceding the survey

^*c*^ n (%) for categorical variables; Median (interquartile range) for continuous variables

^*d*^ *p*-values estimated using Fisher’s exact test or Pearson’s Chi-squared test for categorical variables, or Wilcoxon rank sum test for continuous variables, bold where *p*-value <0.05

^*c*^ *p*-values estimated using Fisher’s exact test for categorical variables or Wilcoxon rank sum test for continuous variables , bold where *p*-value <0.05

More than half of respondents (51.9%, *n* = 110/212) were from three counties (City of Edinburgh (*n* = 42/212, 19.8%), Aberdeenshire (*n* = 37/212, 17.5%), and Highland (*n* = 31/212, 14.6%)), and participants from 10 counties contributed to 81.1% (*n* = 172/212) of responses (Table 2).

**Table 2:** Participants’ primary county of residence and proportion who reported at least one tick bite

		Tick bite in the 12 months preceding survey	
County of residence	**Overall**, *N* = 212 ^*a*^	**No**, *N* = 37^*a*^	**Yes**, *N* = 175^*a*^
Aberdeenshire	37 (17.5%)	5 (13.5%)	32 (86.5%)
Argyll and Bute	4 (1.9%)	0 (0.0%)	4 (100.0%)
City of Aberdeen	9 (4.2%)	2 (22.2%)	7 (77.8%)
City of Edinburgh	42 (19.8%)	6 (14.3%)	36 (85.7%)
City of Glasgow	13 (6.1%)	1 (7.7%)	12 (92.3%)
Dundee	4 (1.9%)	0 (0.0%)	4 (100.0%)
Fife	6 (2.8%)	1 (16.7%)	5 (83.3%)
Highland	31 (14.6%)	4 (12.9%)	27 (87.1%)
Perth and Kinross	7 (3.3%)	1 (14.3%)	6 (85.7%)
Scottish Borders	7 (3.3%)	3 (42.9%)	4 (57.1%)
South Lanarkshire	7 (3.3%)	3 (42.9%)	4 (57.1%)
Stirling	13 (6.1%)	1 (7.7%)	12 (92.3%)
West Lothian	5 (2.4%)	2 (40.0%)	3 (60.0%)
Other county	27 (12.7%)	8 (29.6%)	19 (70.4%)
Study total	**212 (100.0%)**	**37 (17.5%)**	**175 (82.5%)**

^*a*^ n (%)

### Period prevalence of tick bites

Among the 212 hill runners, 82.5% (*n* = 175/212, 95%CI 76.76–87.40) reported having been bitten by at least one tick in the 12 months preceding the survey, and 91.5% (*n* = 194/212, 95%CI 86.91–94.89) reported at least one tick bite in their lifetime prior to the 12-month survey period (Table 1). Of the 212 participants, 24 reported being bitten by a tick outside of Scotland at least once in the 12 months preceding the survey (11.3%, 95%CI 7.39–16.37).

### Medical attention and antibiotic prescription

Of those who reported a tick bite in the 12 months preceding the survey, 10.3% (*n* = 18/175) sought medical attention (pharmacy for treatment, a general practitioner or other hospital-based care), and 8.6% (*n* = 15/175) reported receiving antibiotics (Table 1). All participants who reported a clinical diagnosis of LD or erythema migrans reported receiving a course of antibiotics in response to a tick bite (type and course duration were not recorded).

### Period prevalence and incidence of treated suspected Lyme disease

Of the 212 participants, 4.2% (*n* = 9/212, 95%CI 1.96–7.91) reported treated suspected LD in the 12 months preceding the survey, of whom one reported no identified erythema migrans. The incidence of reported treated suspected LD for the 212 study participants was 4245.3 per 100,000 (95%CI 1941.21-8058.87). No participants reported having had a positive diagnostic test (either by the NHS or privately) for LD in the 12 months preceding the survey.

Argyll and Bute had the highest reported incidence of treated suspected LD at 25000.0 per 100,000 (*n* = 1/4, 95%CI 632.95-139291.10), but the lowest number of participants of areas reporting treated suspected LD diagnoses. This was followed by Highland at 12903.2 per 100,000 (*n* = 4/31, 95%CI 3515.69-33037.38), City of Aberdeen at 11111.1 per 100,000 (*n* = 1/9, 95%CI 281.31-61907.15), City of Edinburgh at 4761.9 per 100,000 (*n* = 2/42, 95%CI 576.69-17201.64), and Aberdeenshire at 2702.7 per 100,000 (*n* = 1/37, 95%CI 68.43-15058.50).

For the lifetime period prior to the 12-month survey period, 8.0% (*n* = 17/212, 95%CI 4.74–12.53, unadjusted for age) of participants reported treated suspected LD, of whom 47.1% (*n* = 8/17, 95%CI 22.98–72.19) reported a positive diagnostic test, all conducted by the NHS. Three participants with treated suspected LD in their lifetime prior to the survey period also had a diagnosis in the most recent 12 months. History of erythema migrans rash prior to the 12 month survey period was not surveyed.

Among the 144 participants who reported taking part in SHR-affiliated races in the preceding 12 months, 4.9% (*n* = 7/144, 95%CI 1.98–9.76) reported a treated suspected LD diagnosis. Assuming conservatively that this represents all LD diagnoses from SHR’s 4668 registered competitors, this results in a minimum treated suspected LD incidence of 150.0 per 100,000 (95%CI 60.29-308.97) among those taking part in SHR-affiliated races.

### Preventive behaviours

**Fig. 1:**
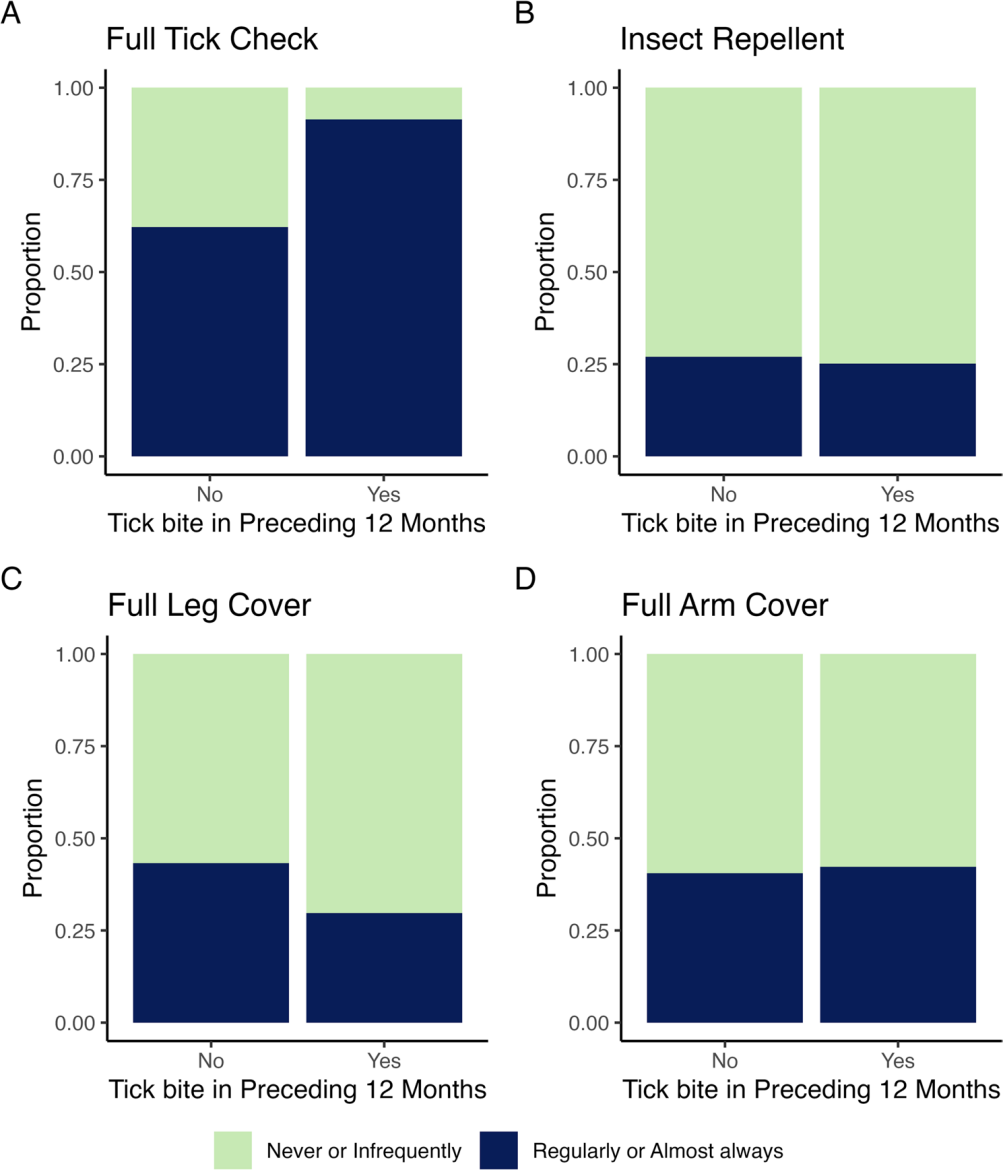
Prevention behaviours by report of at least one tick bite in the 12 months preceding the survey. Prevention behaviours: **A**. full tick check, **B**. insect repellent, **C**, full leg cover, and **D**. full arm cover, are split by report of at least one tick bite in the 12 months preceding the survey, and proportions given for never or infrequently (green) and regularly or almost always (blue) undertaking the prevention behaviour

Most hill runners reported regularly/almost always conducting tick checks (86.3%, *n* = 183/212), but never/infrequently using insect repellent (74.5%, *n* = 158/212), wearing full leg cover (67.9%, *n* = 144/212) or arm cover (58.0%, *n* = 123/212) (Fig. 1; Table 1).

When asked for free-text reasons for not applying NHS recommended tick prevention behaviours, 68.4% (*n* = 145/212) of all survey participants responded that they were too warm to wear full body cover when running (Table 3).

**Table 3:** Summary of participant responses for non-adherence to tick bite prevention behaviours, and prevention methods used when hill running

a. Reported reason for non-adherence with preventive behaviours	*n*	Percentage (%) of total participants^*a*^
Too warm or uncomfortable in body cover	145	68.4%
Not previously thought of tick bites or Lyme disease as a concern to address	20	9.4%
Forget to apply insect repellent, conduct checks or cover up	16	7.5%
Concern for environmental or health impacts of insect repellents	11	5.2%
Not previously aware of tick bite risk or prevention tools available	10	4.7%
Not believed body cover or insect repellent to be effective	8	3.8%
Find it easier to see tick bites on bare skin compared to when wearing body cover	4	1.9%
Expense of insect repellent	2	0.9%
Not sure what to look for with regards to finding tick bites	1	0.5%

b. **Reported other or additional method of tick bite prevention**	**n**	**Percentage (%) of total participants** ^***a***^
Regular checks for tick bites during running activity	25	11.8%
Where possible, avoiding crossing dense vegetation, e.g. bracken and known tick areas	13	6.1%
Tucking trousers, including waterproofs, into socks	8	3.8%
Spraying clothes and shoes with insect repellent treatment, e.g. permethrin	7	3.3%
Wearing long and/or waterproof socks	6	2.8%
Never sitting down or placing items on the ground	5	2.4%
Showering straight away and/or washing off regularly e.g. in rivers	5	2.4%
Keeping legs hair free	4	1.9%
Keeping clothes separate or in sealed bags after running activity	2	0.9%
Use of other chemicals, e.g. Deep Heat, Suncream	2	0.9%

^*a*^ Individuals may have given more than one reason for non-adherence or additional method; these are counted separately

**Fig. 2:**
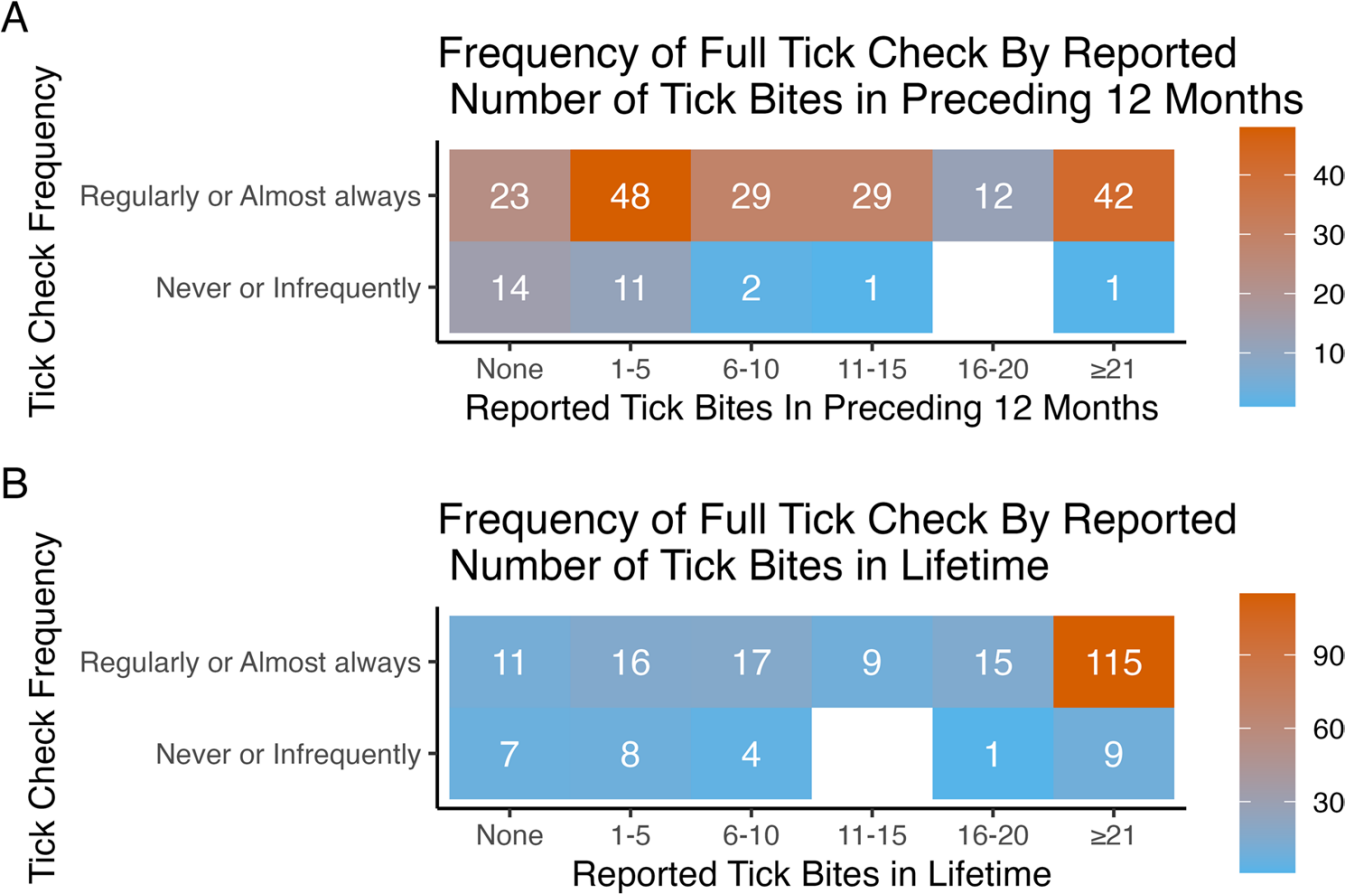
Reported number of tick bites, by frequency of conducting full body tick checks. Reported number of tick bites between March to October when crossing heather, bracken, long grass and/or forestry, in (**A**) the 12 months preceding the survey and (**B**) over the participant’s lifetime prior to the 12-month survey period. Orange represents the highest number of tick bites and blue the lowest

Regularly/almost always conducting a full body tick check, compared with never/infrequently, was associated with proportionally more tick bites over the 12 months preceding the survey (*p* < 0.001, Fig. 2A, Additional file 2), and with more tick bites in the respondents’ lifetime preceding the survey period (*p* < 0.001, Fig. 2B).

**Fig. 3:**
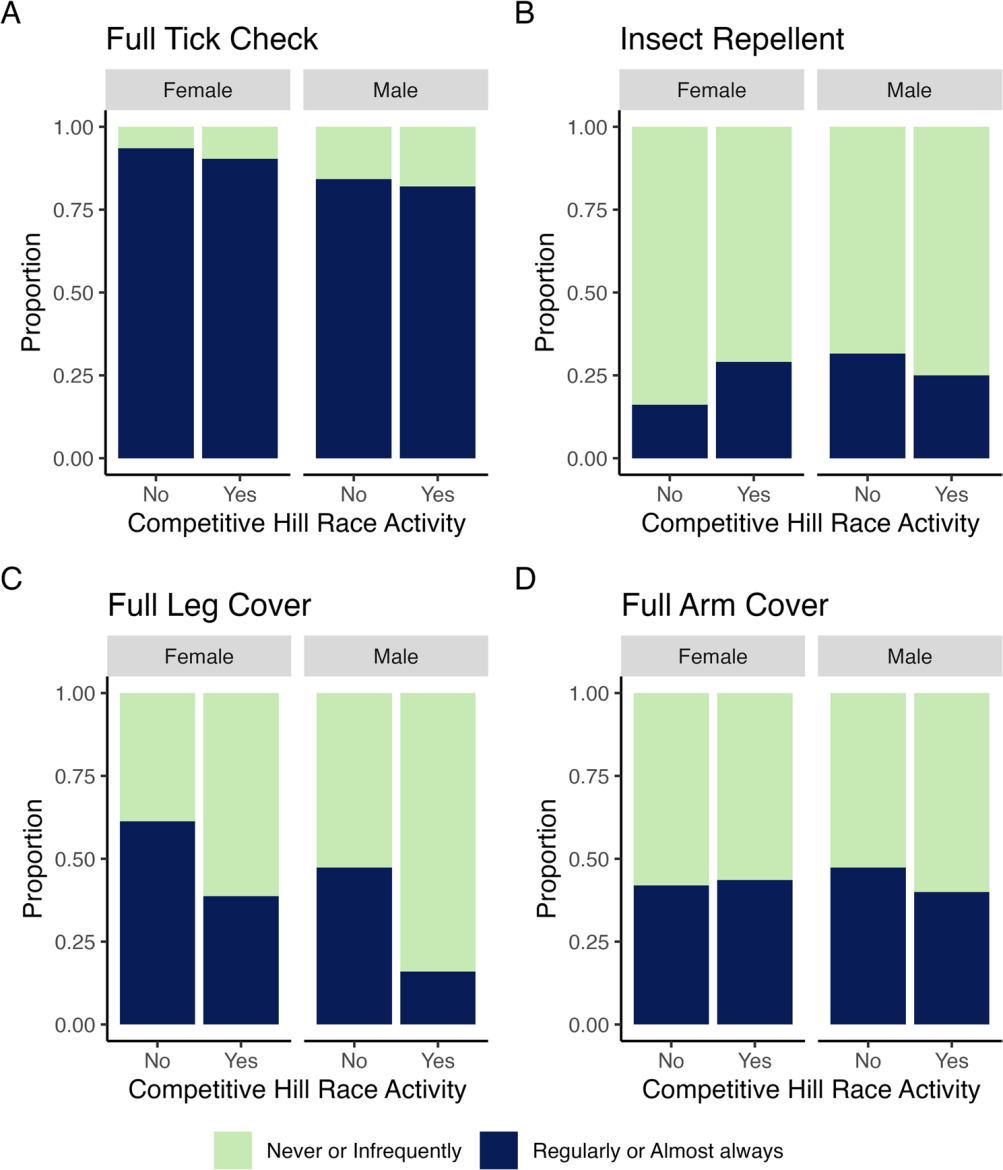
Prevention behaviours, by sex and competitive hill racing status in the 12 months preceding the survey. Prevention behaviours: **A**. full tick check, **B**. insect repellent, **C**, full leg cover, and **D**. full arm cover, are split by sex and competitive hill racing activity (No = non-competitive runners; Yes = competitive hill racers) in the 12 months preceding survey, and proportions given for never or infrequently (green) and regularly or almost always (blue) undertaking the prevention behaviour

The median age of participants who regularly/almost always conducted full body tick checks was younger than those who never/infrequently did (39 vs. 45 years, *p* = 0.036, Additional file 3). A higher proportion of males reported participating in competitive hill races in the preceding 12 months (84.0%, *n* = 100/119 vs. 66.7% for females; *p* = 0.003, Additional file 4), but a greater proportion of females (46.2%, *n* = 43/93) reported regularly/almost always wearing full leg cover when running (compared with 21.0%, *n* = 25/119 of males, *p* < 0.001). Among males, the proportion of competitive hill racers who reported never/infrequently wearing full leg cover was higher (84.0%, *n* = 84/100) than for non-competitive runners (52.6%, *n* = 10/19, *p* = 0.006, Fig. 3).

### Factors associated with tick bites

Regularly/almost always performing full body tick checks (compared to never/infrequent checks) was associated with higher odds of reporting at least one tick bite in the past 12 months (OR 8.52, 95%CI 3.29–22.90, *p* < 0.001). In contrast, regularly/almost always wearing full leg cover (compared with never/infrequently) was associated with lower odds of having at least one tick bite (OR 0.35, 95% CI 0.13–0.89, *p* = 0.029, Table 4).

**Table 4:** Multiple logistic regression analysis of at least one tick bite in the 12 months preceding survey

**Variables**	Overall, N = 206^*a*^	Tick bite in the 12 months preceding survey						
		No, N = 35^*a*^	**Yes**, N = 171^*a*^	OR	95% CI	*p*-value	Average Dominance R^2*c*^	Ranking
Full body tick check							0.094	1
Never or infrequently	29 (14.1%)	14 (40.0%)	15 (8.8%)	1.00	(reference)			
Regularly or almost always	177 (85.9%)	21 (60.0%)	156 (91.2%)	8.52	3.29, 22.90	**<0.001**		
Hours of running (per 1 hour/week increment)	6.0 (4.2, 8.0)	6.0 (4.5, 8.0)	6.0 (4.5, 8.0)	0.99	0.89, 1.13	0.928	0.034	2
Hours of other outdoor activity (per 1 hour/week increment)	5.0 (3.0, 8.0)	5.0 (3.0, 7.0)	5.0 (3.0, 9.0)	1.07	1.00, 1.20	0.116	0.03	3
Full leg cover							0.019	4
Never or infrequently	140 (68.0%)	19 (54.3%)	121 (70.8%)	1.00	(reference)			
Regularly or almost always	66 (32.0%)	16 (45.7%)	50 (29.2%)	0.35	0.13, 0.89	**0.029**		
Sex							0.002	5
Female	90 (43.7%)	14 (40.0%)	76 (44.4%)	1.00	(reference)			
Male	116 (56.3%)	21 (60.0%)	95 (55.6%)	0.81	0.33, 1.97	0.640		
Proportion of running that is hill running							0.001	=6
Never or infrequently	31 (15.0%)	7 (20.0%)	24 (14.0%)	1.00	(reference)			
Regularly or almost always	175 (85.0%)	28 (80.0%)	147 (86.0%)	0.97	0.30, 2.74	0.950		
Insect repellent use							0.001	=6
Never or infrequently	155 (75.2%)	26 (74.3%)	129 (75.4%)	1.00	(reference)			
Regularly or almost always	51 (24.8%)	9 (25.7%)	42 (24.6%)	0.85	0.34, 2.22	0.729		
Full arm cover							0.001	=6
Never or infrequently	119 (57.8%)	20 (57.1%)	99 (57.9%)	1.00	(reference)			
Regularly or almost always	87 (42.2%)	15 (42.9%)	72 (42.1%)	1.13	0.49, 2.68	0.775		
Age (per 1 year increment)	40 (31, 49)	45 (30, 50)	40 (31, 48)	1.01	0.98, 1.05	0.487	0.001	7
Competitive hill racing in preceding 12 months							<0.01	8
No	46 (22.3%)	8 (22.9%)	38 (22.2%)	1.00	(reference)			
Yes	160 (77.7%)	27 (77.1%)	133 (77.8%)	1.00	0.34, 2.79	0.990		

^*a*^ Median (interquartile range) for continuous variables; n (%) for categorical variables

^*b*^ Bold where *p*-value <0.05

^*c*^ R^2^ = McFadden’s Pseudo-R-Squared

Abbreviations: *OR* Odds Ratio, *CI* Confidence Interval

McFadden’s pseudo-R-squared value for the model was 0.18. Among the variables included, conducting a tick check explained the most variance in reporting at least one tick bite (0.094, Table 4, Additional file 5).

## Discussion

This online cross-sectional survey found high period prevalence of reported tick bites and LD among the surveyed Scottish hill running population. Adherence with recommended prevention behaviours was generally low, however most hill runners reported regularly/almost always conducting full body tick checks, and this was associated with higher odds of having at least one tick bite. Regularly/almost always wearing full leg cover was associated with lower odds of having at least one tick bite in the preceding 12 months.

Period prevalence of tick bites in the study population (82.5%) was higher than the prevalence reported from mass participation events, including a Scottish hill running competition (8.5% to 13.8%) [34], and Scottish (33.3%) [17] and French (62.4%) [29] orienteering events. While this is not unexpected given the timeframe studied here, the period prevalence was also higher than that found in comparable general population cross-sectional surveys conducted in 2016 across Denmark, Norway and Sweden (52%, 49.3% and 71.3% respectively) [26]. In addition, the proportion of respondents reporting ≥ 21 tick bites in the preceding 12 months was much higher in our study population (20.3%) than the 1.8% reported by Jore et al. in the Scandinavian general population [26]. A lack of tick bite data in the UK general population and other at-risk groups limits the ability to make comparisons to the wider population, which is further limited by not capturing data on tick bites acquired through occupational and other activities. Nevertheless, this study highlights that taking part in off-road running activity represents a particularly high-risk behaviour for tick bites [26, 27].

The period prevalence of reported treated suspected LD (4.2%) was higher than the 2% reported by Jore et al. [26]. However, unlike that European study, where most individuals had a confirmatory serological test [26], no hill runners in our study reported a diagnostic test to confirm LD diagnosis in the 12-month period. Interpretation and generalisation of both tick bite and LD prevalence calculations for hill runners should be interpreted cautiously due to possible recall bias in the bite date, diagnosis date, the relatively small population studied, and potential motivation bias towards survey participation in those with experience of tick bites or LD, which may inflate the observed prevalence. However, the clinical LD diagnosis definition used here without confirmatory serological testing aligns with NICE guidelines for diagnosis and treatment of LD in the UK, whereby individuals who develop erythema migrans should be prescribed antibiotic treatment without laboratory testing [4]. This is largely because the antibody response takes several weeks to become detectable, limiting the value of serological testing in acute treatment decisions [4]. As a result, none of the treated suspected LD cases reported here would be included in Scottish national surveillance, which reports on serological testing only [2]. This supports the previously acknowledged underestimates of LD incidence reported in Scottish national surveillance [2, 15], which reinforces the case for including erythema migrans reporting as a key indicator in national surveillance [13], since similar studies utilising treated erythema migrans have indicated much higher incidence rates [6, 15, 42]. Nevertheless, a lack of confirmatory testing increases the risk of inappropriate antibiotic use for misdiagnosed infections.

All those presenting with erythema migrans were treated with antibiotics as per NICE guidelines [4] (Table 1). However, 3.0% of those with a tick bite, but no reported rash or LD diagnosis from a clinician, also reported receiving a course of antibiotics following a bite, despite prophylaxis treatment not being a NICE recommendation [4]. However, antibiotics may have been prescribed for a tick bite associated with a localised bacterial skin infection, as the questionnaire did not specifically capture those prescribed for LD. Furthermore, the frequency of erythema migrans presenting in *Borrelia burgdorferi* sensu lato infections and whether this is dependent on other factors is unclear [4]. One treated suspected LD case reported no erythema migrans, but a clinical diagnosis without a positive test, where testing should have taken place. Possibly there was a high clinical suspicion of LD (e.g. due to an atypical rash or other signs and symptoms not captured by the questionnaire) and a prescription made whilst awaiting test results, which aligns with the guidance [4].

The estimated LD incidence in the study population, 4245.3 per 100,000 per year, may be biased towards overestimation due to (i) increased likelihood that runners who had experienced LD completed the questionnaire and (ii) possible inaccuracy in remembering exactly when an LD event occurred. In contrast, the estimated minimum incidence of LD among SHR competitive individuals, 150.0 per 100,000 per year, is likely an underestimate, given the low survey participation rate (3%) by the SHR competitive hill runner population. Nonetheless, both figures are higher than reported previous estimates which take into account all clinically diagnosed LD, including treated erythema migrans with no serological confirmation, of 37.3 per 100,000 (95% CI 34.2–40.7) in the general population of Scotland in 2010–2012 [6]. This suggests hill runners may be at increased risk compared to the general population. However, this difference may also be partially explained by the time period between estimates, changes in vector distribution [20], and changes in human interactions with the environment, such as changes to outdoor leisure activity in general during and after the SARS-COV-2 pandemic [2]. Validating self-reported findings via medical records and improving case definition consistency in research and surveillance initiatives would enable more accurate comparisons in the UK and internationally [12, 13]. Study participants reported regular engagement in other outdoor activities or occupations in addition to hill running (Table 1), which could lead to an overestimation of the incidence related to hill running within the study population. The overall results are closer to recent LD incidence estimates for the general population living in the Scottish Highlands (149–362 per 100,000) [42] and the Western Isles (53–574 per 100,000) [15]. Therefore, it is plausible that the results of this study may simply reflect the high incidence of LD within the general Scottish population. However, the estimated incidence of treated suspected LD for participants from the Highland area in this study was higher than the calculated incidence across the whole study area. This suggests hill runners in the Highland area may be at greater risk of LD than the general population. Hall et al. demonstrated high prevalence of tick bites at individual hill running events and confirmed *Borrelia burgdorferi* presence within ticks biting participants [34], but further long-term study is required to confirm the link between specific bites acquired when hill running and any attributable LD diagnosis. Research to define the hill running population denominators and understand other possible confounding variables is vital for more accurate incidence calculations and comparisons in specific groups, as well as improving surveillance of LD in Scotland.

The higher odds of at least one tick bite being related to conducting a full body tick check has been demonstrated previously [43] and may be a form of detection bias. Searching for ticks may be inherently associated with the number of tick bites found. It is also possible that tick bites were missed by those not conducting checks. Because even an initial full body check may not always be enough to completely detect all, especially small, ticks [29], overall prevalence of tick bites may be underestimated. The association may also be related to previous experience of tick bites, which is supported by the strong relationship between higher numbers of tick bites over a participant’s lifetime and increased frequency of regularly/almost always conducting tick checks. Additionally, associations between specific geographical locations of hill runs and factors such as the degree of exposure, prior education, or risk perception may influence the likelihood of conducting tick checks [43, 44]. However, we were not able to comprehensively investigate these relationships here, although these factors were mentioned by some participants in free-text responses (Table 3).

The overall low adherence with wearing full body cover in hill runners is consistent with the perceived impacts on comfort and performance commented on by most survey respondents and was similarly observed in an earlier mass participation study of hill runners in Scotland [34]. The lower adherence in those engaging in competitive hill running also supports this, as racing is likely to be associated with higher energy output activity (and thus feeling hot/sweating) more regularly. It is unclear why women comply with full leg cover more frequently than men. Any compulsory measure for full leg cover, in line with British orienteering [33], would need to be carefully balanced with other perceived health risks by participants, and be well evidenced, as perception of efficacy has been shown to relate to uptake [44]. This study provides new evidence suggesting that full leg covering is protective for preventing tick bites, supporting previous evidence that body coverage during outdoor activities may reduce the risk of LD [23].

Adherence with insect repellent application was low but was within the range of use reported by orienteers (8.5–34.2%) in multi-day orienteering events [29]. Previous evidence for the effectiveness of insect repellent is of low quality, but a systematic review suggested that use reduces LD incidence rates [23], and more novel repellents have demonstrated efficacy in preventing tick bites in field trials [38]. Individual studies around permethrin-treated body cover clothing options are demonstrating some promise in preventing tick bites in trials with forestry workers. However, indications of limitations with durability of the treatment and inconsistent findings across studies limit the ability to conclude its potential use in other populations, such as hill runners [45–48]. In addition, it is unlikely that permethrin-treated clothing would be an easy recommendation to implement in hill running due to the apprehension to use full body cover. Nevertheless, a small number of participants reported the use of permethrin sprays on other items of clothing (Table 3). This study suggests there is potential to lower the risk of tick bites and LD by improving awareness of risk and promoting uptake of prevention strategies that can be easily adopted and supported within the community. For instance, signposting to information and encouraging prevention in newsletters, social media and at events could help raise awareness. Providing repellents and tick removal tools at registration and aid stations at events could potentially increase protection uptake by those citing a lack of knowledge of risk, forgetfulness or cost.

A major strength of this study is that it rapidly surveyed a geographically dispersed population of hill runners in Scotland and investigated self-reported LD diagnosis over the previous year and lifetime, an area not directly addressed in previous studies of mass participation events [17, 29, 34]. A limitation is that it is possible participants may have incorrectly recalled or mistaken questing ticks, other insects or arachnids for tick bites. Ascertaining photographic evidence to validate tick bite findings would be valuable.

There has never been a more important time to consider the impacts of vector borne diseases to people undertaking hill running, for which participation has risen in recent years in Scotland, in line with the increasing popularity of trail and mountain running internationally. Changing climate and the spread of TBEV within native ticks and host populations of the UK [5, 21, 22, 49], pose heightened dangers to high-risk groups such as hill runners. Climate shift towards warmer and wetter winters extends tick habitat range, phenology and questing season [50]. This includes extension of tick habitat to higher altitudes where previously tick survival was limited [36, 49]. Such conditions give rise to more active ticks and more frequent bites to running and orienteering competitors in Scotland and elsewhere [17, 34]. Taking a One Health approach, which considers the unique interactions between hill runners and other specific at-risk groups, to the changing environment, host abundance, and disease vectors, will be key to understanding changing risk and help to give rise to more effective interventions for reducing LD burden [10, 11, 51]. A longitudinal cohort study utilising medical records to validate LD diagnosis and prescribing in high-risk groups, and recording various exposures, could garner evidence for improving long-term surveillance and prevention of LD. Furthermore, sentinel surveillance of high-risk populations could capture the impacts beyond acute manifestations of LD and other tick-borne diseases.

## Conclusions

This study provides new evidence that hill runners are an at-risk group for tick-borne diseases within Scotland and uncovered potential benefits of prevention behaviour adherence. There is scope to implement effective health protection recommendations to tackle tick-borne diseases, and the support from hill runners in conducting this study demonstrates a motivation to improve evidence and understanding of risk within this community as well as the population more broadly.

## Supplementary Information

Supplementary Material 1.

Supplementary Material 2.

Supplementary Material 3.

Supplementary Material 4.

Supplementary Material 5.

## Data Availability

A summarised dataset will be made available on reasonable request.

## Abbreviations

LD: Lyme disease
TBEV: Tick-borne encephalitis virus
SHR: Scottish Hill Runners
