# Supplementary material for: Running into the Lyme-light: a retrospective cross-sectional study of tick bites and Lyme disease prevalence, incidence, and prevention in hill runners, Scotland, UK
Source: BMC Public Health. 2026 Jan 13;26:528. doi: 10.1186/s12889-025-26181-8 (PMC12888623; doi:10.1186/s12889-025-26181-8)
Supplement: Supplementary file 1 — Supplementary Material 1. [file 12889_2025_26181_MOESM1_ESM.pdf]

Additional File 1. Survey questionnaire data dictionary.

Number	Question	Unit	Answer Type
1	Have you been living in Scotland for the past 12 months?	Yes/No	Binary
2	Is hill running an activity you have participated in during the last 12 months?<br><br>Hill running can be defined as running any distance over hills, moors and mountains.	Yes/No	Binary
3	Have you taken part in a Scottish Hill Runners affiliated race in the last 12 months?<br><br>According to those listed in the Scottish Hill Runners racing calendar which can be found here: www.scottishhillrunners.uk/Calendar.aspx	Yes/No/Not Sure	Binary
4	Have you taken part in a 'hill race' in Scotland not listed by Scottish Hill Runners?	Yes/No/Not Sure	Binary
5	What is your age?		Numerical
6	What is your sex?	Male/Female/Other/Prefer not to say	Multiple choice
7	What county of Scotland has been your primary address for the last 12 months?<br><br>If you are unsure, please find a list and maps of the 32 council authority areas defined by the Scottish government here: www.gov.scot/publications/local-authority-maps-of-scotland/<br><br>If you have lived at multiple addresses within Scotland, select the address you have spent the most months resident in overall.	City of Aberdeen<br>Aberdeenshire<br>Angus<br>Argyll and Bute<br>Clackmannanshire<br>Dumfries and Galloway<br>Dundee<br>East Ayrshire<br>East Dunbartonshire<br>East Lothian<br>East Renfrewshire<br>City of Edinburgh<br>Falkirk<br>Fife<br>City of Glasgow<br>Highland<br>Inverclyde<br>Midlothian	Multiple choice

		Moray<br>North Ayrshire<br>North Lanarkshire<br>Perth and Kinross<br>Renfrewshire<br>Scottish Borders<br>South Ayrshire<br>South Lanarkshire<br>Stirling<br>West Dunbartonshire<br>West Lothian<br>Na h-Eileanan Siar (Western Isles)<br>Orkney Islands<br>Shetland Islands	
8	On average, please estimate the number of **hours per week** you spent running outdoors in the last 12 months.		Numerical
9	Out of the time you have spent running outdoors, please estimate the proportion of time you spent running through heather, bracken, long grass and/or forestry in the last 12 months.	Never/Infrequently (0-35%), regularly (36-65%), almost always (66-100% of the time)	Factorial
10	On average, please estimate the number of **hours per week** of other outdoor activity you have taken part in over the last 12 months.<br><br>Other outdoor activity might include but is not limited to, orienteering, cycling, walking, and hiking.		Numerical
11	Please estimate the total number of tick bites you have had **in the last 12 months**.<br><br>Please only count ticks which have been attached to your skin and which have required removal with a tick removal device or other instrument, and not ticks which have been crawling which could be brushed off.	None, 1-5, 6-10, 11-15, 16-20, > 20	Factorial

12	Out of the total bites you have had **in the last 12 months,** how many of these tick bites were acquired outside of Scotland? (e.g. in England, Wales, NI or abroad)		Free text
13	Please estimate the total number of TICK BITEs you have had in total **in your lifetime**.	None, 1-5, 6-10, 11-15, 16-20, > 20	Factorial
14	Have you sought medical attention for any tick bite **in the last 12 months**?<br><br>Medical attention includes attending a pharmacy for treatment, a general practitioner or other hospital-based care.	Yes, No	Binary
15	Did you develop a ‘bulls-eye’ rash from any tickbites in the last 12 months?<br><br>The definition of a ‘bulls-eye’ rash can be found on NHS Inform: https://www.nhsinform.scot/illnesses-and-conditions/infections-and-poisoning/lyme-disease/	Yes, No	Binary
16	Have you been prescribed any antibiotics for any tick bite or Lyme disease in the last 12 months?	Yes, No	Binary
17	If answering yes to question 16, please let us know what type of antibiotic this was if you remember, and what length of treatment course in days?		Free text
18	Has a healthcare professional given you a clinical diagnosis of Lyme disease at any point in the last 12 months?	Yes, No	Binary
19	Have you received a positive clinical test result for Lyme disease at any point in the last 12 months?	Yes, No	Binary
20	If answering yes to question 19, was this diagnostic test conducted via the NHS or privately?<br><br>If multiple tests have been done over the period, please state your first positive test result.	NHS, Private	Factorial
21	Prior to 12 months ago, have you ever been prescribed antibiotics after a tick bite?	Yes, No	Binary

22	Prior to 12 months ago, has a healthcare professional ever given you a clinical diagnosis of Lyme disease?	Yes, No	Binary
23	Prior to 12 months ago, have you ever received a positive clinical test result for Lyme disease?	Yes, No	Binary
24	If answering yes to question 23, was this diagnostic test conducted via the NHS or privately?<br><br>If multiple tests have been done over the period please state your first positive test result.	NHS, Private	Factorial
25	Have you ever been diagnosed with any other tick-borne disease that you know of? Please provide the disease name and when this was.		Free text
26	Is there any additional information you feel may be relevant to any tick bite, Lyme disease diagnosis, or medical attention you have received? Please provide relevant details.		Free text
27	In the months March to October in the past 12 months when running through heather, bracken, long grass and/or forestry, please estimate how often you use insect repellent.<br><br>E.g. smidge.	Never, infrequently (0-35%), regularly (36-65%), almost always (66-100%)	Factorial
28	In the months March to October in the past 12 months when running through heather, bracken, long grass and/or forestry, please estimate what proportion of the time you wear full leg-covering.<br><br>E.g. full-length leggings or trousers or full-length waterproof covers.	Never, infrequently (0-35%), regularly (36-65%), almost always (66-100%)	Factorial
29	In the months March to October in the past 12 months when running through heather, bracken, long grass and/or forestry, please estimate what proportion of the time you wear full-length arm-covering.<br><br>E.g. a long sleeve top or waterproof jacket.	Never, infrequently (0-35%), regularly (36-65%), almost always (66-100%)	Factorial

30	In the months March to October in the past 12 months when running through heather, bracken, long grass and/or forestry, please estimate what proportion of the time you complete a full-body tick check after your run?	Never, infrequently (0-35%), regularly (36-65%), almost always (66-100%)	Factorial
31	Please describe any additional methods or tools you use to prevent tick bites?		Free text
32	Please describe any reasons why you may **not** follow guidance on using full leg and arm-cover, insect repellent or conducting a full-body tick check.<br><br>Prevention methods can be found on NHS Inform: https://www.nhsinform.scot/illnesses-and-conditions/injuries/skin-injuries/tick-bites/		Free text
