# Supplementary figures and images for: Running into the Lyme-light: a retrospective cross-sectional study of tick bites and Lyme disease prevalence, incidence, and prevention in hill runners, Scotland, UK
Source: BMC Public Health. 2026 Jan 13;26:528. doi: 10.1186/s12889-025-26181-8 (PMC12888623; doi:10.1186/s12889-025-26181-8)

## General Dominance

Predictor

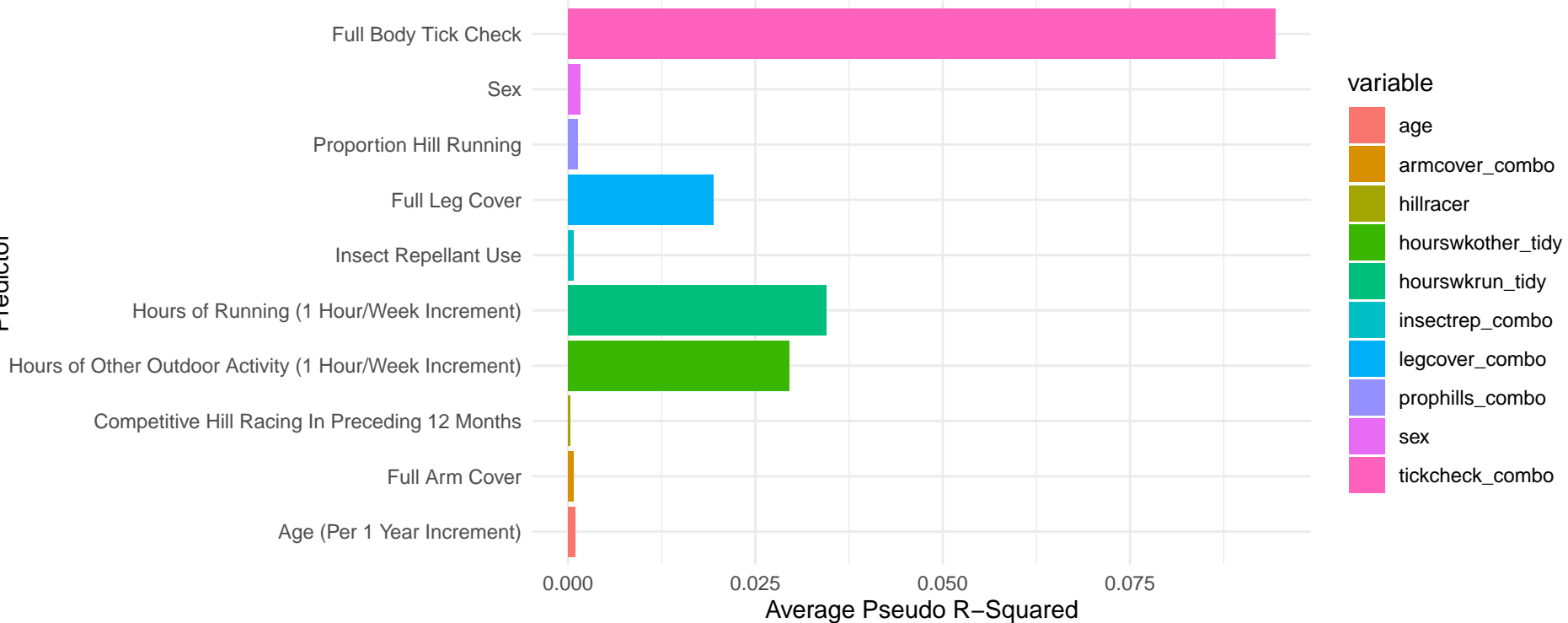

Supplement: Supplementary file 5 — Supplementary Material 5. [file 12889_2025_26181_MOESM5_ESM.pdf]
